# Enhanced detection of sepsis pathogens in hospitalized febrile neutropenia patients using blood culture broths with TaqMan array card

**DOI:** 10.21203/rs.3.rs-9652919/v1

**Published:** 2026-05-22

**Authors:** Furqan Kabir, Hammad Afzal Kayani, Dur e Shahwar, Rufei Ma, Sara Iqbal, Akram Hussain, Jawaid Hussain, Adil Kalam, Fatima Aziz, Sehrish Munir, Farrukh Qazi, Jie Liu, Eric Houpt, Erum Khan, Junaid Iqbal, Asad Ali

**Affiliations:** 1 Department of Pediatrics and Child Health, Aga Khan University, Karachi, Pakistan; 2 Department of Pathology and Laboratory Medicine, Aga Khan University, Karachi, Pakistan; 3 Department of Biosciences, Shaheed Zulfiqar Ali Bhutto Institute of Science and Technology, Karachi, Pakistan; 4 School of Public Health, Qingdao University, Qingdao, China; 5 Division of Infectious Diseases and International Health, School of Medicine, University of Virginia, Charlottesville (VA), USA; 6 Department of Community Health Sciences, Aga Khan University, Karachi, Pakistan

**Keywords:** TaqMan array card, febrile neutropenia, infectious disease, molecular diagnostics

## Abstract

**Background:**

In resource-limited settings, febrile neutropenia (FN) is one of the common cause of morbidity and mortality among immunocompromised individuals particularly those undergoing chemotherapy for malignancies. The clinical diagnosis and management of disease is challenging, due to the diverse range of causative pathogens and overreliance on the traditional diagnostic techniques. TaqMan array card (TAC), a microfluidics-based technique that allows simultaneous and rapid detection of multiple pathogens with high sensitivity, addressing these diagnostic needs.

**Objective:**

The aim of this study was to determine the role of TAC in improving pathogen identification in hospitalized patients with FN.

**Methods:**

This cross-sectional study enrolled 156 FN patients admitted to Aga Khan University and Hospital, Karachi, Pakistan. To enhance pathogen detection, blood culture broths were used for microbial enrichment, improving sensitivity and reliability. Both blood cultures and TAC assays were performed on the collected broths. Enrichment strategies were employed to reduce the impact of PCR inhibitors and increase microbial biomass, thereby enhancing the diagnostic sensitivity of TAC.

**Results:**

TAC identified a diverse range of pathogens compared with traditional blood culture. Of 156 study participants, 61 (39.1 %) were culture-positive and at least one pathogen was detected in 50 of the 146 samples by TAC assay. The common pathogen detected by TAC was *K. pneumonia* followed by *E. coli* and *S. aureus*, and *Candida* species along with two viruses (Parvovirus B19 and HHV6). Kappa values of 0.319 for *E. coli* and 0.644 for *K. pneumoniae*, TAC demonstrated a moderate association with blood culture results. Moreover, *K. pneumoniae*, *E. coli*, and *P. aeruginosa* and *Candida* species were significantly associated with increased mortality among FN patients.

**Conclusion:**

The findings of this study suggest that TAC improves pathogen detection with high sensitivity in FN patients. Incorporating TAC into clinical settings may facilitate early pathogen identification leading to better clinical outcomes. Multicenter studies are needed to validate these results and assess the effectiveness of TAC in broader clinical settings.

## Background

Febrile neutropenia (FN) is a potentially lethal condition commonly affecting immune-compromised patients, particularly those undergoing chemotherapy for malignancies [1]. It is characterized by fever, with an oral temperature ≥ 38.3°C or a temperature of 38.0°C sustained for a minimum of one hour, with a significant reduction in absolute neutrophil count (ANC) i.e. <1.5 × 109/L (1500/mm^3^) [2, 3]. Clinical management is challenging due to impaired innate immunity and chemotherapy-associated mucosal barrier injury, which together predispose patients to infections caused by common pathogen including Gram-negative bacteria (*Escherichia* coli, *Klebsiella* pneumoniae), Gram-positive bacteria (*Staphylococcus* aureus), fungi, and parasites [4]. The clinical manifestation of these infections are often non-specific and progress rapidly, making early detection and pathogen identification crucial for the selection of a suitable antimicrobial regimen and for improving clinical outcomes [5].

The incidence and outcomes of FN vary based on malignancy type, chemotherapy regimen, comorbidities, prophylaxis, and access to healthcare facilities. Globally, FN affect approximately 10–50% of patients suffering with solid tumors and up to 50–70% of patients with hematologic malignancies [6]. In resource limited settings, the incidence maybe higher because of delayed diagnosis and limited supportive care, with a mortality rate ranges from 2–6% in children and can surge up to 50% if antibiotic therapy is not received during the first 48 hours of onset [7, 8]. In Pakistan, there is a dearth of population-level estimates as most of the data are hospital-based with focused on specific subgroups rather than national surveillance. A hospital-based paedriatic prospective cohort from Lahore reported the incidence of 25% of cases monthly in patients undergoing chemotherapy [9] and a tertiary-care hospital of Karachi, Pakistan reported a 5.98% hospital-based incidence of chemotherapy-induced FN [10].

Identification of the causative pathogen in FN is challenging due to nonspecific clinical presentation, heterogeneous etiology, low pathogen biomass, types of specimens, and frequent antecedent antibiotic exposure [11]. Traditional diagnostic methods such as blood culture have long been the gold standard for identifying bacterial infections but their sensitivity is limited by factors such as prior antibiotic use, presences of intracellular, fastidious, and slow-growing bacteria [12]. Molecular methods such as nucleic acid amplification (NAA), particularly multiplex polymerase chain reaction (PCR) can simultaneously detect a wide range of pathogens, reducing the turnaround times. However, blood derived PCR inhibitors such as hemoglobin, lactoferrin, and immunoglobulins can reduce amplification efficiency and can lead to false-negative results particularly in low biomass infections which are common in FN. Several factors can affect the diagnostic sensitivity and reproducibility in FN patients such as variation in nucleic acid extraction, recovery from low input specimens, and recovery of PCR inhibitors [13, 14].

To enhance PCR sensitivity and reliability, allowing more precise pathogen detection in FN, enrichment strategies such as blood culture broth incubation are employed to increase microbial biomass and reduce the impact of PCR inhibitors. [15]. Building on this, the TaqMan array card (TAC), a multiplex real-time PCR based on microfluidics, provides a valuable tool for concurrently identifying 24–384 pathogenic targets. Its ability to analyze a small sample volume of just 1/2 μL combined with constant amplification settings, reliable cycle threshold (Ct) values, and high analytical sensitivity and specificity, makes it a thorough method for the detection of pathogens in FN [15, 16].

In resource-limited settings, such as Pakistan, there is a dearth of information about the etiology of FN, and few studies have specifically addressed practical challenges such as blood-derived inhibition and extraction-related variability. This study aims to assess the effectiveness of TAC in detecting bacterial, viral, fungal, and parasitic targets in patients with FN. To improve diagnostic sensitivity and provide a thorough etiologic profile to improve empiric management and antimicrobial stewardship, blood culture broth incubation and enrichment strategies were employed before nucleic acid extraction and TAC testing.

## Methods

### Study design and participants

A cross-sectional study design was employed, including analysis of blood culture bottles from hospitalized patients with fever and neutropenia. The inclusion criteria were (i) fever, with oral temperature ≥ 38.5 °C at the time of blood collection for broth (ii) the absolute neutrophil count (ANC) of <1500/ml. Patients who had undergone surgery and/or were hospitalized for more than 03 days for illness in the preceding 02 weeks (i.e., within 14 days of last hospitalization) were excluded. The study was approved as an “exemption” and the need for consent to participate was waived by the Ethics Research Committee of Aga Khan University, Pakistan (ERC# 2025-10905-33164), as the study was conducted on leftover/discarded samples with proper de-identification.

### Study setting

This study was conducted at Aga Khan University Hospital (AKUH), a not-for-profit tertiary care academic medical center in Karachi, Pakistan. Blood culture bottles were collected from the clinical and diagnostic laboratory, AKUH between April to November 2025.

### Sample collection and transportation

Blood culture broths were collected, following routine diagnostic procedures and workup from Clinical and Diagnostic Laboratory, AKUH. Samples were transported to Infectious Diseases Research Laboratory (IDRL-AKUH) under cold chain conditions (2–8 °C) and processed on the same day.

### Sample enrichment and concentration

The entire sample volume (avg. 42 ml) from the blood culture bottle was aspirated using disposable 10 ml syringe (BD,) and transfer it to 50 ml conical tubes. Sample concentration was done, using Nanotrap microbiome particle A (mb-A) in combination with enhancement reagent 3 or ER-3 (Ceres Nanosciences, Manassas, VA, USA). Briefly, 1000μl of mb-A and was mixed with 750μl of ER-3 to the sample, which is then incubated for 30 min. at room temperature with periodic mixing after every 5 min to facilitate the enrichment process. The supernatant was carefully removed, using a dyna magnetic (ThermoFisher Scientific, USA) and retained the pellet for subsequent washing steps with nanotrap buffer-2 (Ceres Nanosciences, Manassas, VA, USA) followed by removal of the supernatant using magnetic rack. Finally, the pellet was dissolved in 250 μL of DNA/RNA shield (Zymo Research, USA). Stabilized pellets were processed in batches within 3 days of collection [17].

### Total nucleic acid (TNA) extraction

Total nucleic acid (TNA) was extracted following modified MagMAX ultra nucleic acid isolation kit (ThermoFisher Scientific, USA) protocol. Briefly, the pellets obtained during enrichment and concentration step were mixed with MagMAX lysis buffer and spiked with extrinsic controls MS2, PhHV (to monitor the recovery of RNA and DNA targets respectively). The resulting suspension was then incubated at 95°C for 10 minutes, followed by magnetic separation and ProteinaseK digestion. TNA was captured, using magMAX magnetic binding beads, followed by sequential washing steps with wash buffers and ethanol. Finally, beads were eluted using 200 μL elution buffer. An additional step of PCR inhibitors removal was performed, using OneStep PCR inhibitor removal kit (Zymo Research, USA) as per the manufacturers’ recommendations. TNA was stored at −80°C in an ultra-low temperature freezer, until it was processed for TAC.

### TaqMan Array Card (TAC) processing for pathogens detection

The TAC was customized for 56 pathogens known to cause sepsis or sepsis like illness, including 23 bacteria, 28 viruses, 03 parasites and 02 fungi (see supplementary material for TAC layout). TAC assay was performed as described previously [18–20]. Briefly the reaction mixture was prepared, using TNA and AgPath-ID One-Step RT-PCR Master Mix (Thermo Fisher Scientific, USA), while the QuantStudio 7 Flex platform (Applied Biosystems, Thermo Fisher Scientific) was used for thermal cycling and data generation. A Ct value of ≤35.00 was used to define positivity. Each assay on the TAC was considered as valid only if (i) Extraction and PCR blanks remained negative for that assay/gene target. (ii) MS2 and PhHV remained positive within samples for the validity of RNA and DNA based targets respectively.

### Statistical Analysis

Descriptive statistics are presented as median (M) and interquartile range (Q_1_–Q_3_) for continuous variables and as counts (n) and percentages (%) for categorical variables. Between-group comparisons were performed using the Mann–Whitney U test (Z) for continuous non-parametric data and Pearson’s chi-square test (χ^2^) or Fisher’s exact test for categorical data. Univariable logistic regression was used to evaluate associations between pathogen positivity or clinical variables (Age, WBC, neutrophil count, and ANC) and clinical outcome (Deceased = 1, Discharged = 0), with variables with p < 0.10 entered multivariable logistic regression to calculate adjusted odds ratios (ORs) and 95% confidence intervals (CIs). Spearman correlation was applied to assess associations between pathogens and laboratory markers. Associations with leukemia diagnosis were analyzed using chi-square or Fisher’s exact test, and ORs were calculated for 2×2 tables where appropriate. All analyses were performed in R (tidyverse, readr, broom) with two-sided p < 0.05 considered statistically significant.

## Results

### Clinical characteristics

A total of 156 blood cultures were performed, of which 147 blood cultured broth samples were further processed and tested using TAC. Among the 147 patients, the median age was 21.5 (7.0, 48.8) years with majority (88, 60.3%) being male (Table 1; placed at the end of the manuscript). The patients were admitted to 16 different wards, while PAEDS-ONCO (30.8%), HAEM-ONCO (24.0%), and ONCO (19.2%) collectively account for approximately three-quarters of admissions. The clinical diagnosis for these patients included febrile neutropenia (64, 43.8%), leukemia (27, 18.5%), septic shock (12, 8.2%), and dengue (6, 4.1%), as shown in Table 2. 32 (21.9%) patients were deceased. The deceased patients were older than those discharged (P=0.013), and they were mostly from HAEM-ONCO, ONCO, PAEDS-ICU, and NICU (P<0.001). Most of the deceased patients were diagnosed as septic shock (10, 31.3%), compared with 1.8% (2/114) among discharged patients (P<0.001).

### Pathogen detection with blood culture

Among the 156 samples, 61 (39.1%) were positive for at least one type of blood culture. For the three types of blood culture, 88, 74, and 66 samples were tested, yielding 33 (37.5%), 21 (28.4%), and 25 (37.9%) positive results for aerobic, anaerobic, and paedriatic culture, respectively. *Klebsiella* species were the most prevalent (13, 8.3%), with 12/13 identified as *Klebsiella pneumonia*. *E. coli* and *Pseudomonas aeruginosa* were the second most detected at 7.7% (12/156), followed by *Staphylococcus* species (10, 6.4%) with 2/10 identified as *Staphylococcus* aureus. Other less prevalent pathogens detected included *Acinetobacter* spp. (5, 3.2%), *Enterococcus* spp. (4, 2.6%), Candida tropicalis (3, 1.9%), *Corynebacterium* spp. (2, 1.3%), *Enterobacter* spp. (2, 1.9%), *Stenotrophomonas* maltophilia (2, 1.3%), *Serratia* spp. (2, 1.3) with one identified as *Serratia* marcescens, *Burkholderia* cepacian complex (1, 0.6%), *Campylobacter* spp. (1, 0.6%), *Plesiomonas* shigelloides (1, 0.6%), *Pseudomonas* stutzeri (1, 0.6%), *Streptococcus* group D (1, 0.6%), and *Streptococcus* mitis (1, 0.6%). Significantly higher culture positivity was observed for deceased patients (26/35, 74.3%) than that of discharged patients (34/120, 28.3%), with 68.2% (15/22) versus 21.7% (12/60) for aerobic, 68.8% (11/16) versus 11.3% (6/53) for anaerobic, and 70.0% (7/10) versus 28.3% (15/53) for paedriatic culture (P<0.05).

### Pathogen detection with TAC

At least one pathogen was detected in 50 of the 146 samples, with 32 had a single pathogen identified, 15 had two, and 3 had three. As shown in Table 3, K. pneumoniae (24/146, 16.4%) were the predominate bacterium detected by TAC, followed by *E. coli* (13/146, 8.9%) and an *S*. aureus (13/146, 8.9%). Other bacteria detected included *P*. aeruginosa (5/146, 3.4%), A. baumannii (4/146, 2.7%), and S. pneumoniae (1/146, 0.7%). Interestingly, two viruses were identified, with Parvovirus B19 (4/146, 2.7%) and HHV6 (1/16, 0.7%).

For the most detected *K. pneumoniae* and *E. coli*, TAC showed moderate correlation with the original culture results, yielding Kappa values of 0.644 and 0.319, respectively. The culture positive samples showed lower Cq values than those of culture negative but TAC positive.

### Pathogen association with mortality

The pathogen association with mortality was further explored (Table 5 a, b). The univariate analysis showed that age was a risk factor for mortality (OR 1.02, 95% CI 1.00–1.04). *E. coli*, *K. pneumoniae*, and *P. aeruginosa* were strongly associated with mortality, with OR being 4.6 (1.2, 18.1), 5.1 (1.9, 14.1), and 10.7 (1.6, 90.5) respectively.

The multivariate analysis (Table 6 a, b) confirmed these association trends for all three pathogens with Cq cutoff at both 40 and 35, particularly K. pneumoniae (OR 4.4, P=0.040) while with inconsistent statistical significance for E. coli (OR 3.7, P=0.063), and P. aeruginosa (OR 7.5, P=0.056).

No significant correlation was observed for pathogen detection with the infection biomarkers such as white blood cell counts and absolute neutrophil counts as presented in Table 7 (placed at the end of the manuscript).

## Discussion

In this study, we assessed the effectiveness of TAC for improved pathogen identification in hospitalized patients suffering from FN, a disease that significantly increases morbidity and mortality in immuno-compromised individuals. The findings highlight the high-risk nature of FN among patients receiving chemotherapy, particularly those with hematological malignancies. Mortality was higher among older patients and those admitted to intensive care units, hematologic oncology, and neonatal intensive care wards, showing the increased vulnerability of these patients. These findings are consistent with previous studies, indicating advanced age and comorbid conditions such as leukemia as risk factors for FN [21].

Septic shock, resulting from multiple organ dysfunction due to dysregulated inflammatory response, was the leading cause of death among patients with FN receiving chemotherapy [22]. To prevent septic shock and improve clinical outcomes, the precise identification of causative agent is required to ensure appropriate antibiotic therapy. Prior studies emphasized significance of targeted antibiotic therapy guided by microbiological identification in reducing the mortality rates in FN patients, specifically those who are at a higher risk of severe infections [23, 24]. The most prevalent pathogen identified by blood culture was *Klebsiella pneumoniae* (39.1%), followed by *Escherichia coli* and *Pseudomonas aeruginosa*. These findings are consistent with the results of previous studies showing *E. coli* and *K. pneumonia* as major contributors of bacterial infections in FN patients, especially those with hematological malignancies or receiving chemotherapy [25]. The substantially higher culture positivity observed in deceased patients (74.3%) compared with those discharged (28.3%) reflects the high positivity of these pathogens in advanced FN, indicating a clear correlation between culture-confirmed infections and unfavorable outcomes. According to previous studies, *P. aeruginosa* and other multi-drug-resistant pathogens have been linked to higher mortality in FN patients [23, 24]. This highlights the importance of rapid pathogen identification and tailored antimicrobial therapy [26, 27]. Lower culture positivity rates in discharged patients indicate that negative or low-density cultures represent a limitation of blood cultures in FN, emphasizing the need for more sensitive diagnostic techniques to ensure timely and accurate pathogen detection.

A more diverse range of pathogens was detected by TAC compared with blood culture, with at least one pathogen detected in 50 out of the 146 samples, *Klebsiella pneumoniae* was the most prevalent pathogen, followed by *Escherichia coli* and *Staphylococcus aureus*. Although Staphylococcus was identified, it must be considered that many other non-S. aureus species of Staphylococcus considered contaminants in the absence of predisposing factors, such as long-term catheterization or implanted medical devices. Therefore, future studies are required to evaluate clinical significance of non-S. aureus Staphylococcus species in the context of FN.

Additionally, Candida species were also detected, which are clinically significant in immunocompromised patients, particularly those with FN. Conventional blood culture techniques often fail to detect Candida, particularly in patients with prior antibiotic usage, highlighting the role of TAC in identifying such non-culturable organisms. The inclusion of Candida in the list of detected pathogens emphasizes the importance of using molecular diagnostics to complement traditional methods, offering a more comprehensive view of the pathogen landscape in febrile neutropenia. Our findings are supported by a previous study conducted by Ahmed et al., in which 13.6% of blood sample were culture-positive using BACT/Alert blood culture system. Among the positive cases, Gram-negative bacteria were predominant (72.1%), with *Salmonella* typhi being the most prevalent isolated organism (36.9%) [28]. In addition to these pathogens, TAC also detected a wide range of viral pathogens, including *Parvovirus B19* and *HHV-6*. The correlation between TAC and blood culture showed a moderate correlation for *K. pneumoniae* (Kappa = 0.644) and *E. coli* (Kappa = 0.319), as detailed in Table 4 b, indicating that although TAC is more sensitive in identifying a broad spectrum of pathogens, discrepancies may occur owing to low biomass of pathogens, prior antibiotic usage, and the detection thresholds. Although some PCR results were considered false positives when compared with blood culture, they might not be false, because they were clinically significant based on patient mortality rate.

Previous studies showed molecular techniques including TAC as a sensitive technique for the detection of low biomass infections. These techniques can detect pathogens that may not grow in culture due to the fastidious nature of organisms or the prior use of antibiotics [29]. TAC showed 71 % sensitivity compared with blood culture in a study for acute febrile illness (AFI) outbreak investigation and surveillance [30]. In immunocompromised individuals, TAC serves as valuable tool for rapid and specific pathogen detection since viral pathogens are mostly overlooked by traditional culture techniques [31]. However, none of the FN patients in our study underwent organism-specific serological testing, such as ELISA, or individual real-time PCR. The correlation between TAC and blood culture results showed notable discrepancies. In cases with TAC positive and culture negative results, indicate TAC’s potential in detecting low-biomass pathogens or viruses like Parvovirus B19, which traditional blood culture may fail to identify.

However, cases with TAC negative and culture positive results highlight that blood culture is more precise at detecting pathogens that grow well in culture, particularly bacteria that may not reach the required detection threshold for TAC. The cases with both TAC negative and culture positive results highlight the limitations of both diagnostic methods, particularly in cases when biomass is low, or the symptoms have noninfectious origins.

*P. aeruginosa*, *E. coli*, and *K. pneumoniae* were found to be associated with high mortality. Multivariable analysis identified a significant correlation between K. pneumoniae and a higher risk of mortality (OR 4.4, P = 0.040). This finding align with the study by Ceken et al.’s study, which identified pneumonia and advanced age as major risk factors for mortality in patients with FN [32]. No significant association was found between pathogen identification and traditional infection biomarkers such as white blood cell count and absolute neutrophil count. This suggests that these biomarkers are nonspecific and unreliable for diagnosing FN. Advanced molecular diagnostics techniques, including TAC, can improve diagnostic yield by identifying multiple pathogens simultaneously.

For the diagnosis of FN, TAC can be used in conjunction with blood culture, enhancing the detection of a wide range of pathogens, particularly those that are challenging to culture or present in low biomass. TAC is a valuable complementary tool for identifying viral and fastidious pathogens that may be missed by traditional culture. In the future, a more targeted panel might focus on the pathogens that are most prevalent in FN cases, improving diagnostic processes, speed and accuracy of pathogen identification.

The study findings offer valuable insights, however, certain limitations should be considered. The study was conducted on a small number of hospitalized patients with FN from a single hospital (single-center study), limiting the generalizability of the results. Second, we couldn’t determine whether our study has a lesser turnaround time than culture, since we used blood culture bottles followed routine diagnostic workup and this could be the reason that certain pathogens (particularly viruses) might be undetected. A future multicenter study with defined blood culture incubation time points is needed.

## Conclusion

The study aimed to determine the effectiveness of blood culture and TAC in detecting causative pathogens in hospitalized patients with FN. TAC showed high sensitivity, particularly for co-infections*. Klebsiella pneumoniae*, *E. coli*, and *P. aeruginosa*, and *Candida* species were associated with increased mortality. These findings support the potential of TAC as a complement to traditional diagnostic techniques by providing rapid and precise pathogen detection, ultimately improving clinical outcomes.

## Supplementary Files

This is a list of supplementary files associated with this preprint. Click to download.

- SupplementaryFileTACLayoutFK08May2026.pptx

## Figures and Tables

**Table 1:** Demographics, clinical characteristics, and blood culture results of TAC-tested patients (n = 146), stratified by outcome (Deceased vs Discharged).

Variables	Total (n = 146)	Deceased (n = 32)	Discharge (n = 114)	Statistic	*P*
Age (year), M (Q_1_, Q_3_)	21.50 (7.00, 48.75)	41.00 (15.00, 58.00)	18.00 (2.37, 40.75)	Z=−2.49	**0.013**
Age group, n(%)				-	**0.017**
<5	35 (23.97)	6 (18.75)	29 (25.44)		
5–14	20 (13.70)	1 (3.12)	19 (16.67)		
15–49	57 (39.04)	12 (37.50)	45 (39.47)		
50–69	28 (19.18)	9 (28.12)	19 (16.67)		
≥70	6 (4.11)	4 (12.50)	2 (1.75)		
Gender (M/F), n(%)				χ^2^=0.08	0.771
F	58 (39.73)	12 (37.50)	46 (40.35)		
M	88 (60.27)	20 (62.50)	68 (59.65)		
WBC, M (Q_1_, Q_3_)	0.80 (0.50, 2.18)	0.50 (0.50, 1.52)	0.80 (0.50, 2.20)	Z=−1.19	0.233
Neu, M (Q_1_, Q_3_)	14.00 (5.47, 25.87)	11.00 (5.00, 26.20)	15.00 (6.60, 25.80)	Z=−0.40	0.687
ANC, M (Q_1_, Q_3_)	154.40 (41.62, 403.50)	131.00 (39.20, 465.00)	154.80 (42.00, 372.00)	Z=−0.14	0.885
Ward, n(%)				-	**<.001** *
PAEDS-ONCO	45 (30.82)	2 (6.25)	43 (37.72)		
HAEM-ONCO	35 (23.97)	12 (37.50)	23 (20.18)		
ONCO	28 (19.18)	6 (18.75)	22 (19.30)		
MED-WARD	9 (6.16)	1 (3.12)	8 (7.02)		
PAEDS-MED	6 (4.11)	0 (0.00)	6 (5.26)		
MED-IN	5 (3.42)	1 (3.12)	4 (3.51)		
PAEDS-ICU	4 (2.74)	3 (9.38)	1 (0.88)		
NICU	3 (2.05)	3 (9.38)	0 (0.00)		
DAYCARE-ONCO	2 (1.37)	0 (0.00)	2 (1.75)		
ICU	2 (1.37)	1 (3.12)	1 (0.88)		
PAEDS MED-IN	2 (1.37)	0 (0.00)	2 (1.75)		
GEN-SURGERY	1 (0.68)	1 (3.12)	0 (0.00)		
MED-ICU	1 (0.68)	1 (3.12)	0 (0.00)		
PAEDS	1 (0.68)	0 (0.00)	1 (0.88)		
PAEDS-CICU	1 (0.68)	1 (3.12)	0 (0.00)		
PULMONARY-IN	1 (0.68)	0 (0.00)	1 (0.88)		
Blood culture results-Aerobic (pos/neg/bpcn), n(%)				-	**<.001**
bpcn	1 (1.22)	1 (4.55)	0 (0.00)		
neg	53 (64.63)	6 (27.27)	47 (78.33)		
pos	28 (34.15)	15 (68.18)	13 (21.67)		
MISS	64 (43.84)	10 (31.25)	54 (47.37)		
Blood culture results-Anaerobic (pos/neg/bpcn), n(%)				χ^2^=18.85	**<.001**
neg	52 (75.36)	5 (31.25)	47 (88.68)		
pos	17 (24.64)	11 (68.75)	6 (11.32)		
MISS	77 (52.74)	16 (50.00)	61 (53.51)		
Blood culture results-Paeds (pos/neg/bpcn), n(%)				χ^2^=4.73	**0.03**
neg	41 (65.08)	3 (30.00)	38 (71.70)		
pos	22 (34.92)	7 (70.00)	15 (28.30)		
MISS	83 (56.85)	22 (68.75)	61 (53.51)		
Blood Culture Bottles Pooled/Not pooled, n(%)				χ^2^=0.02	0.899
Not pooled	79 (54.11)	17 (53.12)	62 (54.39)		
Pooled	67 (45.89)	15 (46.88)	52 (45.61)		

Z: Mann-Whitney test, χ^2^: Chi-square test, -: Fisher exact, *: Simulated p-value; M: Median, Q_1_: 1st Quartile, Q_3_: 3st Quartile

**Table 2:** Primary clinical diagnoses and leukemia subtypes among TAC-tested patients, stratified by clinical outcome (Deceased vs Discharged) (n = 146)

Variables	Total (n = 146)	Deceased (n = 32)	Discharge (n = 114)
Febrile Neutropenia, n(%)	64 (43.84)	7 (21.88)	57 (50.00)
Dengue, n(%)	6 (4.11)	0 (0.00)	6 (5.26)
Septic Shock, n(%)	12 (8.22)	10 (31.25)	2 (1.75)
Leukemia, n(%)	27 (18.49)	4 (12.50)	23 (20.18)
Leukemia type, n(%)			
Acute Myeloid Leukemia	2 (1.37)	0 (0.00)	2 (1.75)
Acute Myeloid Leukemia (Standard Risk)	1 (0.68)	0 (0.00)	1 (0.88)
Acute Myeloid Leukemia, ( t 8:21 ).	1 (0.68)	0 (0.00)	1 (0.88)
AML	1 (0.68)	0 (0.00)	1 (0.88)
Early precursor T cell Acute Lymphoblastic			
Leukemia / Mucormycosis	1 (0.68)	0 (0.00)	1 (0.88)
ETP - ALL	1 (0.68)	0 (0.00)	1 (0.88)
Late Relapse B-cell Acute Lymphoblastic Leukemia	1 (0.68)	0 (0.00)	1 (0.88)
Newly diagnosed Burkitt Leukemia (Stage 4)	1 (0.68)	0 (0.00)	1 (0.88)
Relapse Acute Myeloid Leukemia	1 (0.68)	1 (3.12)	0 (0.00)
Relapse B-cell ALL	1 (0.68)	0 (0.00)	1 (0.88)
Secondary acute myeloid leukemia	1 (0.68)	1 (3.12)	0 (0.00)
Very early relapse T-cell ALL	1 (0.68)	1 (3.12)	0 (0.00)

**Table 3 (a):** Pathogen positive rates detected by TaqMan Array Card (TAC) using CT < 40 (n = 146).

Positive rate by pathogen (CT<40)			
Pathogen	Total	Positive n	Positive rate
*E.coli*	146	33	0.23
*K. pneumoniae*	146	24	0.16
*S.aureus*	146	13	0.09
*P.aeruginosa*	146	5	0.03
*A.baumannii*	146	4	0.03
*ParvovirusB19*	146	4	0.03
*S.pneumoniae*	146	2	0.01
HHV6	146	1	0.01

**Table 3 (b):** Pathogen positive rates detected by TaqMan Array Card (TAC) using CT < 35 (n = 146).

Positive rate by pathogen (CT<35)			
Pathogen	Total	Positive n	Positive rate
*E.coli*	146	13	0.09
*K.pneumoniae*	146	11	0.08
*A.baumannii*	146	4	0.03
*P.aeruginosa*	146	4	0.03
*S.aureus*	146	2	0.01
*ParvovirusB19*	146	1	0.01
*S.pneumoniae*	146	1	0.01

Positive n = number of samples positive for the pathogen; Positive rate = Positive n / Total.

**Table 4 (a):** Agreement between TaqMan Array Card (TAC) and blood culture for pathogen detection using CT < 40 (n = 146).

Analysis of consistency between TAC and blood culture (CT<40)								
Pathogen	TP	FN	FP	TN	Kappa_value	Sensitivity	Specificity	McNemar.s.test.P.value
*P.aeruginosa*	1	6	4	145	0.134	0.143	0.973	0.7518
*E.coli*	7	1	26	122	0.282	0.875	0.824	0
*K. pneumoniae*	4	2	20	130	0.219	0.667	0.867	**3.00E-04**
*A.baumannii*	1	2	3	150	0.27	0.333	0.98	1
*Candida*	0	1	0	155	0	0	1	1
*E.faecalis*	0	3	0	153	0	0	1	0.2482
*S.aureus*	0	2	13	141	−0.023	0	0.916	**0.0098**

**Table 4 (b):** Agreement between TaqMan Array Card (TAC) and blood culture for pathogen detection using CT < 35 (n = 146).

Analysis of consistency between TAC and blood culture (CT<35)								
Pathogen	TP	FN	FP	TN	Kappa_value	Sensitivity	Specificity	McNemar.s.test.P.value
*P.aeruginosa*	1	6	3	146	0.154	0.143	0.98	0.505
*E.coli*	7	1	6	142	0.644	0.875	0.959	0.1306
*K. pneumonia e*	3	3	8	142	0.319	0.5	0.947	**2.28E-01**
*A.baumannii*	1	2	3	150	0.27	0.333	0.98	1
*Candida*	0	1	0	155	0	0	1	1
*E.faecalis*	0	3	0	153	0	0	1	0.2482
*S.aureus*	0	2	2	152	−0.013	0	0.987	1

* McNemar’s test P < 0.05 indicates a statistically significant disagreement between the test method and the reference standard

TP = true positive; FN = false negative; FP = false positive; TN = true negative; Kappa_value = Cohen’s kappa; Sensitivity = TP / (TP + FN); Specificity = TN / (TN + FP). McNemar’s test P < 0.05 indicates statistically significant disagreement between TAC and blood culture results.

**Table 5 (a):** Univariable and multivariable logistic regression analyses for association between pathogen detection and mortality (TAC CT < 40; n = 146).

univariate+multivari logistic analysis (CT<40) Univariate analysis					
Variable	Type	OR	CI_lower	CI_upper	P_value
*K.pneumoniae*	Pathogen	5.1	1.9	14.1	**0.001**
Age (year)	Clinical	1.0	1.0	1.0	**0.013**
*P.aeruginosa*	Pathogen	10.7	1.6	90.5	**0.015**
*S.aureus*	Pathogen	2.0	0.5	7.2	0.301
*S.pneumoniae*	Pathogen	4.4	0.2	120.6	0.308
WBC	Clinical	1.0	1.0	1.0	0.380
ANC	Clinical	1.0	1.0	1.0	0.482
Neu	Clinical	1.0	1.0	1.0	0.590
*E.coli*	Pathogen	1.1	0.4	2.9	0.825
*A.baumannii*	Pathogen	98055446.5	1.24E-67	NA	0.989
*ParvovirusB19*	Pathogen	3.44E-07	NA	3.32E+52	0.990
*HHV6*	Pathogen	6.09E-07	NA	3.73E+122	0.992

**Table 5 (b):** Univariable and multivariable logistic regression analyses for association between pathogen detection and mortality (TAC CT < 35; n = 146).

univariate+multivari logistic analysis (CT<35) Univariate analysis					
Variable	Type	OR	CI_lower	CI_upper	P_value
*Age (year)*	Clinical	1.0	1.0	1.0	**0.013**
K.pneumoniae	Pathogen	4.6	1.2	18.1	**0.024**
*E.coli*	Pathogen	4.4	1.8	16.2	**0.024**
*P.aeruginosa*	Pathogen	7.0	0.8	64.6	0.068
*WBC*	Clinical	1.0	1.0	1.0	0.380
ANC	Clinical	1.0	1.0	1.0	0.482
Neu	Clinical	1.0	1.0	1.0	0.590
A.baumannii	Pathogen	98055446.5	1.2398E-67	NA	0.989
*S.aureus*	Pathogen	1.2514E-06	NA	9.1759E+63	0.989
*ParvovirusB19*	Pathogen	1.0483E-06	NA	5.474E+122	0.992
*S.pneumoniae*	Pathogen	1.07E-06	NA	5.75E+122	0.992

**Table 6 (a):** Multivariable logistic regression for mortality with TAC CT < 40 (n = 146).

Multivariate analysis					
Variable	Type	OR	CI_lower	CI_upper	P_value
*K. pneumoniae*	Pathogen	4.3	1.5	11.9	**0.005**
*P.aeruginosa*	Pathogen	7.0	0.9	62.7	0.054
'Age (year)'	Clinical	1.0	1.0	1.0	0.063

**Table 6 (b):** Multivariable logistic regression for mortality with TAC CT < 35 (n = 146).

Multivariate analysis					
Variable	Type	OR	CI_lower	CI_upper	P_value
*K.pneumoniae*	Pathogen	4.4	1.0	18.6	**0.040**
*'Age (year*)'	Clinical	1.0	1.0	1.0	**0.044**
P.aeruginosa	Pathogen	7.4	0.8	68.1	0.056
*E.coli*	Pathogen	3.6	0.8	14.3	0.063

Variables with P < 0.10 in univariable analysis were considered for inclusion in the multivariable logistic regression model

**Table 7 (a):** Spearman correlation between pathogen detection and clinical biomarkers (WBC, Neu, ANC) using TAC CT < 40 (n = 146).

correlation analysis between Pathogen and Clinical indicators (CT<40)			
Pathogen	Variable	Correlation	P_value
A.baumannii	WBC	−0.109630347	0.187752684
A.baumannii	Neu	−0.06873708	0.416321798
A.baumannii	ANC	−0.083079072	0.325631254
E.coli	WBC	0.039153864	0.638918541
E.coli	Neu	0.030858954	0.715437263
E.coli	ANC	0.039086532	0.644203562
HHV6	WBC	−0.086996949	0.296423543
HHV6	Neu	−0.02569755	0.76145917
HHV6	ANC	−0.052421022	0.535538593
K.pneumoniae	WBC	−0.100215809	0.22876889
K.pneumoniae	Neu	0.073624045	0.383885305
K.pneumoniae	ANC	−0.00848594	0.920161994
P.aeruginosa	WBC	−0.082621778	0.3214718
P.aeruginosa	Neu	0.018654172	0.825602839
P.aeruginosa	ANC	−0.029845548	0.72439896
ParvovirusB19	WBC	0.07498302	0.368384994
ParvovirusB19	Neu	0.04571665	0.5890286
ParvovirusB19	ANC	0.090390872	0.284708068
S.aureus	WBC	−0.087436671	0.293979577
S.aureus	Neu	0.04666335	0.581329545
S.aureus	ANC	0.020395131	0.809622563
S.pneumoniae	WBC	0.028322883	0.734343697
S.pneumoniae	Neu	0.01896512	0.822743191
S.pneumoniae	ANC	0.044493409	0.59904497

**Table 7 (b):** Spearman correlation between pathogen detection and clinical biomarkers (WBC, Neu, ANC) using TAC CT < 35 (n = 146).

correlation analysis between Pathogen and Clinical indicators (CT<35)			
Pathogen	Variable	Correlation	P_value
A.baumannii	WBC	−0.109630347	0.187752684
A.baumannii	Neu	−0.06873708	0.416321798
A.baumannii	ANC	−0.083079072	0.325631254
E.coli	WBC	−0.15857159	0.05591828
E.coli	Neu	0.040173745	0.635010916
E.coli	ANC	−0.050060776	0.554090685
K.pneumoniae	WBC	0.040936983	0.623711187
K.pneumoniae	Neu	0.113502122	0.178647908
K.pneumoniae	ANC	0.085846805	0.309716251
P.aeruginosa	WBC	−0.109630347	0.187752684
P.aeruginosa	Neu	−0.043119113	0.610388418
P.aeruginosa	ANC	−0.094027286	0.265696014
ParvovirusB19	WBC	0.132030663	0.11215471
ParvovirusB19	Neu	0.017474334	0.836473788
ParvovirusB19	ANC	0.112037087	0.184356853
S.aureus	WBC	−0.123458721	0.137644338
S.aureus	Neu	−0.137132408	0.103657373
S.aureus	ANC	−0.151715232	0.071488098
S.pneumoniae	WBC	0.126913195	0.126886348
S.pneumoniae	Neu	0.013362726	0.874586919
S.pneumoniae	ANC	0.098674865	0.242682776

**Table 7 (c):** Summary of correlation coefficients and P values for pathogen detection versus infection biomarkers (WBC, Neu, ANC) across CT thresholds (n = 146).

Correlation			
Pathogen	ANC	Neu	WBC
*A.baumannii*	−0.083	−0.069	−0.110
*E.coli*	−0.050	0.040	−0.159
K.pneumoniae	0.086	0.114	0.041
*P.aeruginosa*	−0.094	−0.043	−0.110
*ParvovirusB19*	0.112	0.017	0.132
*S.aureus*	−0.152	−0.137	−0.123
*S.pneumoniae*	0.099	0.013	0.127
P_value			
Pathogen	ANC	Neu	WBC
*A.baumannii*	0.326	0.416	0.188
*E.coli*	0.554	0.635	0.056
K.pneumoniae	0.310	0.179	0.624
*P.aeruginosa*	0.266	0.610	0.188
*ParvovirusB19*	0.184	0.836	0.112
*S.aureus*	**0.071**	0.104	0.138
*S.pneumoniae*	0.243	0.875	0.127

Correlation coefficients (ρ) and two-sided P values are shown for each pathogen and clinical variable. Red shading indicates positive correlations, blue shading indicates negative correlations; color intensity reflects the magnitude of the correlation.

## Data Availability

The datasets used and/or analyzed during the current study are available from the corresponding author on reasonable request.

## List of abbreviations

FN: Febrile neutropenia
ANC: Absolute neutrophil count
TAC: TaqMan Array Card
NAA: Nucleic acid amplification
PCR: Polymerase chain reaction
